# The 1918 influenza pandemic in New York City: age-specific timing, mortality, and transmission dynamics

**DOI:** 10.1111/irv.12217

**Published:** 2013-12-02

**Authors:** Wan Yang, Elisaveta Petkova, Jeffrey Shaman

**Affiliations:** Department of Environmental Health Sciences, Mailman School of Public Health, Columbia UniversityNew York, NY, USA

**Keywords:** Age-specific mortality, cross-immunity, effective reproductive number, influenza pandemic, transmission dynamics

## Abstract

**Background:**

The 1918 influenza pandemic caused disproportionately high mortality among certain age groups. The mechanisms underlying these differences are not fully understood.

**Objectives:**

To explore the dynamics of the 1918 pandemic and to identify potential age-specific transmission patterns.

**Methods:**

We examined 1915–1923 daily mortality data in New York City (NYC) and estimated the outbreak duration and initial effective reproductive number (*R*_e_) for each 1-year age cohort.

**Results:**

Four pandemic waves occurred from February 1918 to April 1920. The fractional mortality increase (i.e. ratio of excess mortality to baseline mortality) was highest among teenagers during the first wave. This peak shifted to 25- to 29-year-olds in subsequent waves. The distribution of age-specific mortality during the last three waves was strongly correlated (*r* = 0·94 and 0·86). With each wave, the pandemic appeared to spread with a comparable early growth rate but then attenuate with varying rates. For the entire population, *R*_e_ estimates made assuming 2-day serial interval were 1·74 (1·27), 1·74 (1·43), 1·66 (1·25), and 1·86 (1·37), respectively, during the first week (first 3 weeks) of each wave. Using age-specific mortality, the average *R*_e_ estimates over the first week of each wave were 1·62 (95% CI: 1·55–1·68), 1·68 (1·65–1·72), 1·67 (1·61–1·73), and 1·69 (1·63–1·74), respectively; *R*_e_ was not significantly different either among age cohorts or between waves.

**Conclusions:**

The pandemic generally caused higher mortality among young adults and might have spread mainly among school-aged children during the first wave. We propose mechanisms to explain the timing and transmission dynamics of the four NYC pandemic waves.

## Introduction

An estimated 50 million infected persons died during the 1918 ‘Spanish flu’ pandemic,^1^ making it the deadliest influenza pandemic on record. The massive death toll and public health significance of the event continue to motivate study of its transmission dynamics and impact. It is hoped that improved understanding of the etiology, epidemiology, and repercussions of the 1918 pandemic will better inform preparedness for future influenza pandemic events.

The epidemiological characteristics of the 1918 pandemic were unusual, and the mechanisms responsible for these observed patterns are still not well understood.^2^,^3^ Many regions experienced three waves of the pandemic within the same year, and for some regions, there was only a brief quiescent interval between the second and the third waves, which has not been well explained.^2^,^3^ Additionally, the demographic structure of morbidity and mortality is of interest. The 1918 pandemic disproportionally killed young adults, while school-aged children and the elderly were relatively unaffected.^4^ Other influenza pandemics have also produced shifts in the age structure of infections, as compared with patterns observed for seasonal influenza, including a diminution of elderly infections; however, the apparent higher mortality rate in young adults (20- to 40-year-olds) manifest during the 1918 pandemic has not been recorded since this event.^3^ Whether differences in transmissibility among age groups may have contributed to these patterns has not been examined in detail.

To explore the dynamics of the 1918 pandemic and to identify potential age-specific transmission patterns, we examined daily mortality data for age-stratified cohorts during 1918–1920 in New York City (NYC). Four pandemic waves were evident in NYC from February 1918 to April 1920. For each wave, we identified the onset and ending and calculated the total pandemic-related mortality of each 1-year age cohort. Furthermore, to examine potential differences in transmissibility among age groups, we calculated the effective reproductive number (*R*_e_) for each age (1-year interval) for all pandemic waves. We report the findings here and close with a discussion on the transmission dynamics of the pandemic and possible underlying mechanisms.

## Methods

### Mortality data

We obtained historical daily mortality data from the Genealogy Federation of Long Island. Death certificates between 1915 and 1923 in all NYC boroughs (Bronx, Brooklyn, Manhattan, Queens, and Staten Island) were scanned with permission and entered into a database with careful proofreading and validation. Mortality of all persons 1 year and older was included in the analysis. More details are available in the Appendix S1.

### Identifying the onset and ending for each pandemic wave

Mills *et al*.^5^ defined the initial period of the 1918 pandemic (2nd wave) as the first 3 weeks with excess pneumonia and influenza (P&I) mortality greater than one per 100 000 population. Due to a lack of detailed demographic data, which would have provided a credible denominator (i.e. age-specific populations), we were unable to adopt the same definition for each 1-year age cohort. Instead, to capture the earliest pandemic signal and account for the mortality difference among age groups, we looked for daily mortality in excess of a prescribed threshold. A number of potential thresholds were tested (see Appendix S1); ultimately, we adopted a threshold that approximates the 90% quantile of mortality for each calendar day in interpandemic years. Mortality data in years 1915–1917 (pre-pandemic) and 1921–1923 (post-pandemic) were used as the baseline (interpandemic) years; given the limited duration of the baseline data (6 years) and the existence of extreme values within this record, the 90% quantile was defined as 120% of the second highest baseline mortality record for each calendar day. This threshold was also validated by application to the entire NYC population; excess daily mortality on the onset of each wave (i.e. 51, 70, 106, and 59 excess mortality, for a population of 5·6 million in NYC^6^) was comparable to the threshold adopted by Mills *et al*.^5^

Based on the mortality time series for the entire NYC population, there were four pandemic waves occurring roughly within the following periods: 2/15 to 6/1/1918 (1st wave), 8/1 to 12/2/1918 (2nd wave), 12/3/1918 to 4/30/1919 (3rd wave), and 12/1/1919 to 4/30/1920 (4th wave). These mortality increases can reliably be attributed to pandemic influenza, as other epidemiological studies have identified similar waves^7^,^8^ and molecular studies have confirmed that the pandemic strain was in circulation during early 1918.^9^ Within each of these periods, for each 1-year age cohort, we searched for the first 7 consecutive day timespan with mortality exceeding the estimated 90% quantile threshold level (i.e. onset) and the final day of the last 7 consecutive day timespan with mortality exceeding the same threshold (i.e. ending). This objective search defined the endpoints and duration of each pandemic wave for each 1-year age cohort. All days between the onset and ending were then included as part of that cohort's age-specific pandemic wave. Due to noise in the daily mortality data, daily age-specific mortality can intermittently drop below the threshold, especially during the early phase of a pandemic wave. To account for this noise, we relaxed our definitions of onset and ending to allow 1 day (stricter threshold) or 2 days (looser threshold) among 7 not to exceed the threshold.

### Total excess mortality

Previous studies have used median mortality^5^ or a Serfling regression curve^8^,^10^,^11^ during interpandemic years as a baseline for computing levels of excess mortality. Due to more random noise in the daily data used here, as opposed to weekly or monthly data, we found the former approach more suitable for this study. Per Mills *et al*.,^5^ total pandemic-attributable mortality was defined as the sum of mortality during a pandemic wave minus median daily baseline mortality summed for the same calendar period. Although the dates of onset and ending, as described above, varied with age group, we used a simpler, single longer period for each pandemic wave: 2/27/1918 to 5/30/1918 (1st wave), 8/30/1918 to 12/2/1918 (2nd wave), 12/03/1918 to 4/26/1919 (3rd wave), and 12/03/1919 to 4/24/1919 (4th wave). These ‘broader’ intervals were simply the earliest onset and latest ending detected among all age cohorts for each wave and were used to capture all pandemic-related death.

### Fractional mortality increase and age patterns

To facilitate comparison among age cohorts, we divided total pandemic mortality for each age cohort by cumulative baseline (i.e. median) mortality during the same calendar period. This measure of fractional total mortality increase accounts for different baseline mortality rates among age classes within the population. We calculated this in two ways. In the first, total mortality for a pandemic wave was summed over the calendar period specific to that 1-year age cohort based on the stricter threshold and then divided by the cumulative baseline mortality for the same period. This method allowed for examination of the most intense epidemic episode for each age. Alternatively, we summed the mortality and then divided by the cumulative baseline mortality over entire pandemic episode (i.e. the aforementioned ‘broader’ dates). Using these broader dates, this second method captures sporadic deaths occurring outside each identified intense pandemic period. However, because the denominator includes more cumulative baseline mortality, fractional mortality increase calculated this second way was generally lower than when calculated by the first method.

### Effective reproductive number (*R*_e_) for each pandemic wave

We estimated *R*_e_ for each of the four pandemic waves. For the first and the second waves, *R*_e_ is essentially the basic reproductive number (*R*_0_). We assumed that the pandemic proceeded in a way that can be modeled by a susceptible–infected–recovered (SIR) model, such that the increase in new cases is given by


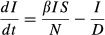
(1)

where *I* is the number of infected, *S* is the number of susceptibles, *N* is the total population, β is the transmission rate, *D* is the infectious period, and *t* is time.

At the beginning of each wave, a certain portion of the population was uninfected (i.e. *S* = *x N*). For the first and second waves, the population was largely naïve, that is, *x* ≈ 1. Substituting for *S*/*N* in Eqn. 1 and then integrating yields:


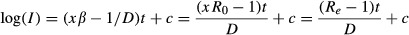
(2)

where *c* is an integration constant. Mortality (*M*) is assumed to scale with *I* with a fixed case-fatality rate (CFR), that is,


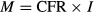
(3)

Combining Eqns 2 and 3 yields:


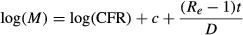
(4)

Based on Eqn. 4, for each age cohort, we fitted daily mortality during the first 7 days of each wave with time to obtain the slope [i.e. (*R*_e_−1)/*D*] of the exponential period of the epidemic curve. The infectious period has been reported ranging from ˜2 to ˜4 days.^5^,^12^ We calculated *R*_0_ or *R*_e_ for *D* equals 2 and 4 days.

## Results

### Four pandemic waves in NYC

**Figure 1:**
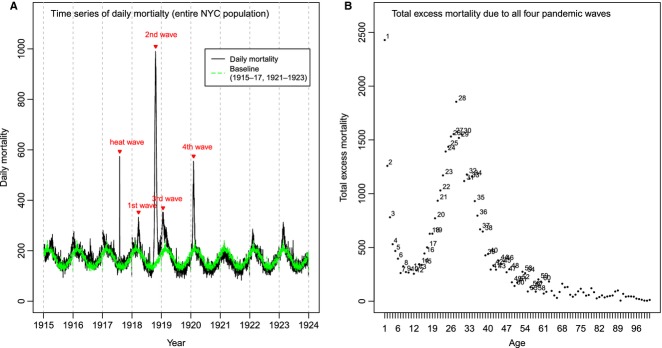
Daily mortality time series for the entire population in New York City in years 1915–1923 (A) and total excess mortality due to all four pandemic waves for each 1-year age cohort (B) Total excess mortality was computed by subtracting the baseline mortality and then summed over the four pandemic periods [i.e. 2/27/1918 to 5/30/1918 (1st wave), 8/30/1918 to 12/2/1918 (2nd wave), 12/03/1918 to 4/26/1919 (3rd wave), and 12/03/1919 to 4/24/1919 (4th wave)]. The numbers associated with the data points are ages at the time of each pandemic episode.

**Figure 2:**
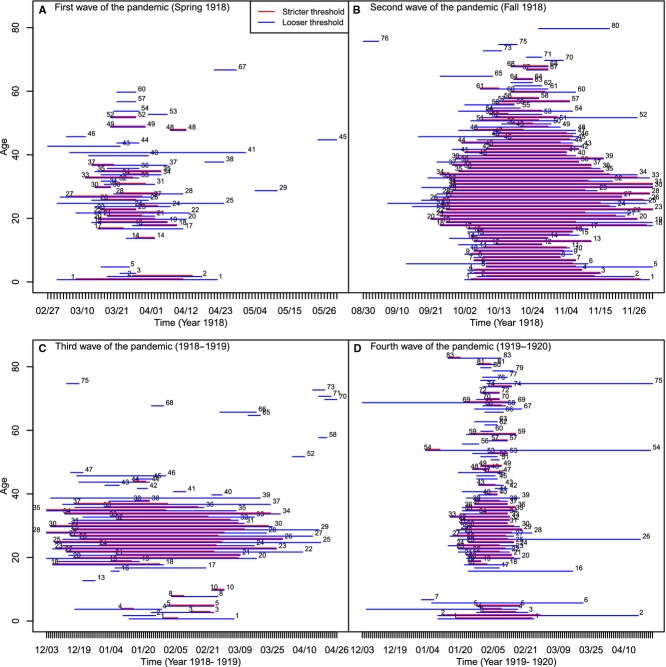
Calendar periods of the four pandemic waves. Labels on the *x*-axis are dates (mm/dd). The numbers associated with the end of each segment are ages at the time of each pandemic episode.

There appear to have been four pandemic waves in NYC throughout the years 1918–1920 (Figure 1A). The calendar durations of each pandemic episode for each age are plotted in Figure 2. An estimated total of 41 188 people, or ˜0·7% of the NYC population, died due to the pandemic. This estimate is slightly higher but consistent with estimates reported by Olson *et al*.^8^ based on monthly mortality records and the Serfling method (40 500 deaths from February 1918 to March 1920). Mortality by age group is summarized in Table 1. Overall, the 1- to 3- and 18- to 38-year age cohorts saw the most mortality during the pandemic (Figures 1B and S1).

**Table 1:** Total excess mortality attributable to each of the four waves of pandemic in New York City NYC (1918–1920)

	1st wave			2nd wave			3nd wave			4th wave			Entire pandemic		
Age	Total	%	FMI (%)*	Total	%	FMI (%)	Total	%	FMI (%)	Total	%	FMI (%)	Total	%	FMI (%)
1–4	827	21·82	53·11	2163	9·90	251·80	646	7·04	30·61	1362	21·37	65·54	4998	12·13	75·69
5–14	328	8·66	54·39	1609	7·36	384·93	718	7·82	84·16	514	8·06	61·31	3168	7·69	116·86
15–19	295	7·78	87·28	1322	6·05	536·11	579	6·31	107·23	343	5·38	64·72	2538	6·16	153·45
20–29	884	23·33	65·29	7463	34·15	721·06	3336	36·37	155·90	1510	23·68	71·73	13 192	32·03	198·88
30–39	541	14·28	29·31	5607	25·66	386·26	2359	25·71	81·76	1110	17·41	39·00	9616	23·35	106·51
40–49	410	10·81	17·52	1897	8·68	99·32	711	7·75	18·76	177	2·78	4·73	3194	7·75	27·12
50–59	201	5·29	7·06	838	3·83	35·86	378	4·12	8·14	260	4·08	5·68	1676	4·07	11·64
60+	305	8·04	5·63	956	4·37	21·64	448	4·88	4·84	1099	17·24	12·02	2807	6·82	9·94
Total	3790	100	23·27	21 853	100	172·68	9172	100	34·99	6374	100	24·65	41 188	100	50·83

**Figure 3:**
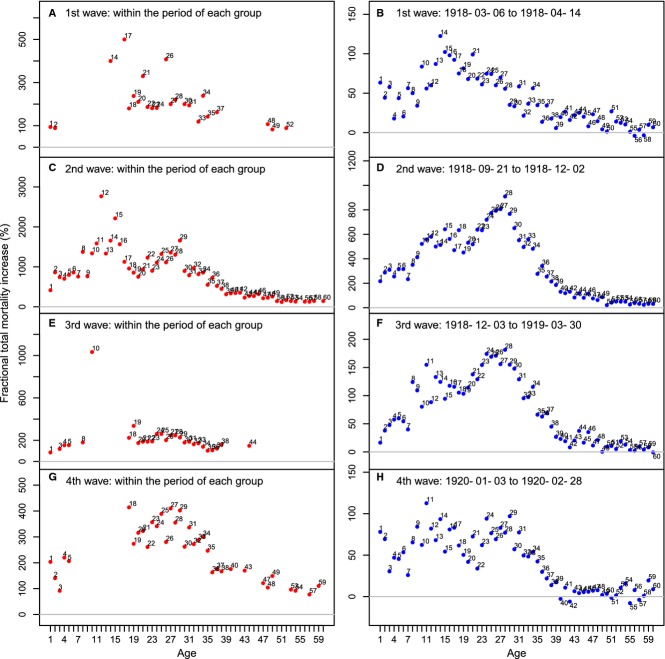
Age-specific mortality patterns in the four pandemic waves. Fractional total mortality increase is the ratio of total excessive mortality to total baseline mortality, either over the pandemic period specific to each age cohort (left panel) or the broader pandemic period for all age groups (specified in each figure, right panel). The numbers associated with the data points are ages at the time of each pandemic wave.

* FMI is the fractional mortality increase, calculated as the ratio of excess mortality to baseline mortality; these values were calculated over the broader pandemic periods corresponding to the right panel in Figure 3; the total FMI (last row) was calculated for the entire NYC population; therefore, it is not a sum or average over all age groups.

Substantial excess mortality was first recorded from 3/6 to 4/14/1918. Assuming a 7–10-day lag between infection and death,^13^ introduction of the pandemic might have occurred during the final week of February, shortly after the decline of seasonal influenza activity that winter. As shown in Table 1, very young children (<5 years olds) and young adults (20–39 years) had the most mortality during the first wave, accounting for over 50% of total mortality.

The second pandemic wave affected most of the NYC population (<˜60 years). Excess mortality occurred approximately from 9/21 through 12/2/1918; an estimated total of 21 853 people died during this wave. Mortality occurred predominantly among young adults with 34·15% of deaths occurring among 20- to 29-year-olds and 25·66% among 30- to 39-year-olds. In addition, these young adults appeared to experience a longer second wave (Figure 2B). This longer duration may in part explain the greater increase in total mortality for these groups, as compared to the first wave (by a factor of ˜10 versus ˜3–6 in other groups, Table 1).

Although most age cohorts (<˜60 years of age) had two mortality peaks from September 1918 to March 1919, some cohorts (e.g. the 28-year-olds) experienced increased mortality throughout the whole period without a clear division between the second and third waves (Figures S3 and S4). We thus set the date with the lowest mortality for the entire NYC population (i.e. 12/2/1918) as the point of division between these two waves. The third wave persisted until 3/30/1919; about 9172 people died during this event.

The fourth wave occurred during the 1919–1920 regular wintertime influenza season. Mortality increased by a factor of ˜2 over baseline (Figure 1A). In addition, increased mortality was observed only during a brief period from 1/3 to 2/28/1920 and most heavily impacted the 20- to 29-, 30- to 39-, and 1- to 4-year-olds (Figure 2D and Table 1).

### Age-specific mortality patterns

To examine mortality patterns in detail, we calculated the fractional increase in excess mortality with respect to the baseline for each 1-year age cohort. Fractional mortality increases calculated using age-specific pandemic periods identify the most intense mortality increase among the age cohorts (left panel of Figure 3). For instance, the fractional mortality increase for ˜10- to 18-year-olds was much greater than for other groups when defined in this fashion. These dramatic mortality increases could be due to a clustering of infections over a short duration (e.g. transmission within a school). Some cohorts, specifically the ˜5- to 16-year-olds, only appear in the second wave, because their increased mortality occurred sporadically during the first wave rather than within a concentrated period (i.e. ≥6 days, Figures 2 and S2–S5). The 1- to 4-year-olds do not exhibit the highest fractional mortality increase, as this age cohort has a high baseline mortality (i.e. the denominator).

The right panel in Figure 3 shows the fractional mortality increase for each ‘broader’ pandemic episode. Despite the ˜5- to 16-year-olds having only sporadic increased daily mortality, consistent with Olson *et al*.,^8^ we found that this age group had the greatest fractional mortality increase during the first wave (Figure 3B). Given their lower CFR,^7^,^14^ these results suggest higher attack rates among the ˜5- to 16-year-olds, especially during the first wave (see more discussion in Appendix S1). The greatest fractional mortality increase shifted toward young adults upon the second wave, as attack rates became comparable among 5- to 34-year-olds in the following waves.^7^,^14^

**Figure 4:**
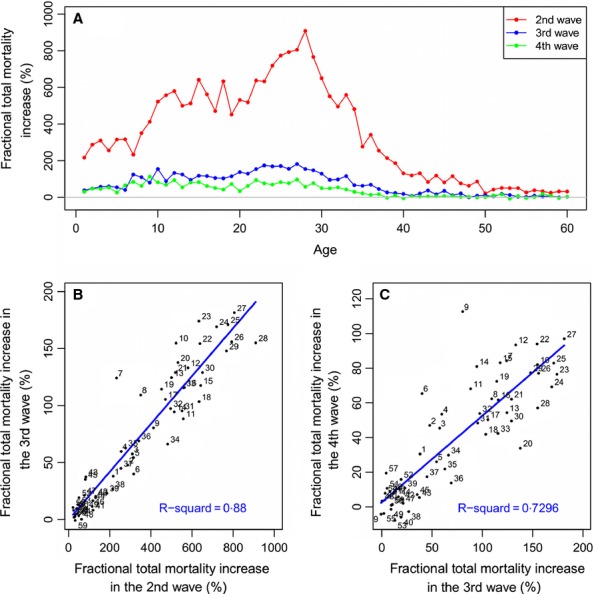
Correlations of age-specific mortality patterns in the final three pandemic episodes. The relative total mortality increase is the same as in Figure 3. The numbers associated with the data points are ages in 1918.

The age patterns of the last three pandemic waves are strikingly similar. In particular, peak mortality occurs among 25- to 29-year-olds (albeit less distinctly during the 4th wave). This similarity is illustrated in Figure 4A, in which the three episodes are superimposed with ages adjusted to the year 1918. It is further evident from Figure 4B, C that 20- to 29-year-olds had the highest fractional mortality increase throughout the final three waves. Other age groups that experience increased fractional mortality include teenagers, the early thirties, young children, and the late thirties. People >50 years of age were largely unaffected.

Previous studies found higher CFR for people under one year and twenty to forty based on US Public Health Service household surveys conducted during 1918–1919.^7^,^14^ As shown in Figure 4B, C, the age-specific mortality patterns in the last three pandemic episodes are strongly correlated, with correlation coefficients of *r* = 0·939 between the second and third waves and *r* = 0·857 between the final two waves. It thus appears that the age cohorts that suffered the greatest mortality early in the pandemic continued to suffer the most. The correlations of age-specific mortality patterns among the second through fourth waves thus to some extent reflect the higher CFR among young adults.

### Transmission characteristics (reproductive number)

**Figure 5:**
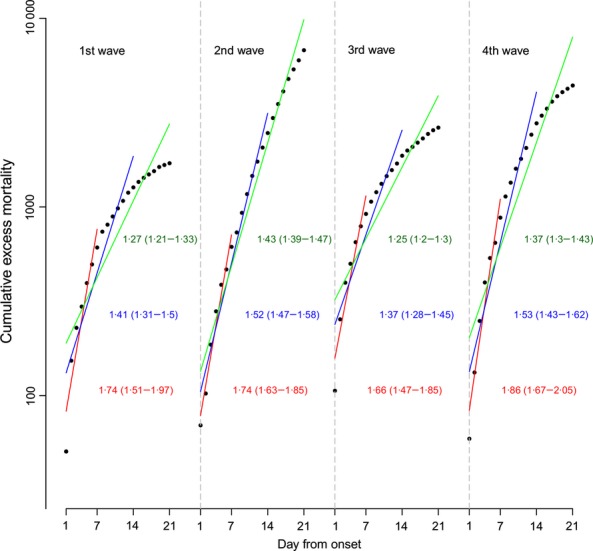
Development of the 1918 pandemic among the entire New York City population. Black dots are cumulative excess mortality at the first 3 weeks of each wave. Lines are fitted with cumulative excess mortality versus days from onset over the first 7 (in red), 14 (in blue), and 21 (in green) days. Color-coded numbers associated with each line are *R*_e_ estimates for each period.

The reproductive number is the average number of secondary cases that arise from one primary case. It reflects the transmissibility of the culprit pathogen and the growth rate of an epidemic. The effective reproductive number (*R*_e_) typically fluctuates over time. To examine the change in pandemic growth rate, we calculated *R*_e_ over the first 1, 2, and 3 weeks of each of the four pandemic waves for the entire NYC population. As shown in Figure 5, *R*_e_ declined with time as transmission persisted beyond the onset of each pandemic wave. For instance, assuming a mean infectious period (*D*) of 2 days, *R*_e_ decreased from 1·74 (1 week) to 1·52 (2 weeks) and 1·43 (3 weeks) during the most severe second wave. The decline in *R*_e_ was more rapid in later waves (Figure 5). However, surprisingly, *R*_e_ at the very beginning of each wave was comparable; *R*_e_ during the first week of each of the four waves was 1·74 (95% CI: 1·51–1·97), 1·74 (1·63–1·85), 1·66 (1·47–1·85), and 1·86 (1·67–2·05), respectively. This similarity of *R*_e_ for the *first week* of each wave is curious. It may reflect initial growth of each wave in a new, previously unexposed subpopulation within NYC in which susceptibility had remained high. In subsequent weeks, as awareness of the outbreak grew and municipal control measures were implemented (e.g. banning of large public gatherings, closing of theaters, churches and schools, and mandated rapid burial of the dead^15^), the growth of each outbreak slowed (i.e. *R*_e_ declined).

**Figure 6:**
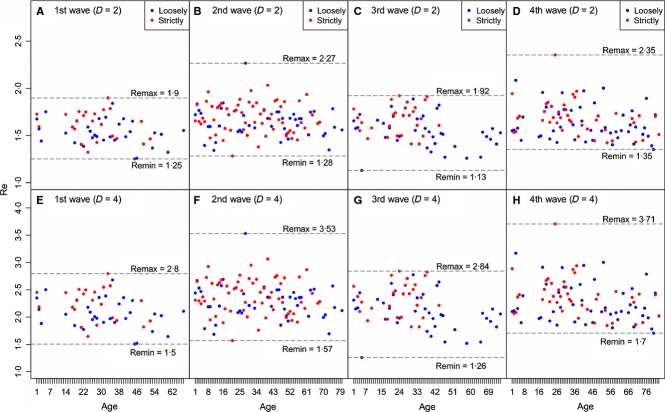
Effective reproductive numbers for the four pandemic waves. These *R*_e_ estimates assume a linearized SIR model and an exponential growth rate over the first 7 days of each pandemic wave, and restricted transmission within an age cohort. *R*_e_s in the upper panel assume an infectious period (*D*) of 2 days, and those in the lower panel assume *D* = 4 days. Red dots are estimates based on the stricter-onset definition, and blue dots are based on the looser-onset definition.

To test potential differences in transmissibility among age groups, we calculated *R*_e_ for each age during the first 7 days of each pandemic wave (Figure 6). For simplicity, *R*_e_ was calculated assuming transmission restricted within an age cohort. Using the stricter threshold definition, the *R*_e_ for each of the four pandemic waves was, respectively, 1·62 (95 CI: 1·55–1·68), 1·68 (1·65–1·72), 1·67 (1·61–1·73), and 1·69 (1·63–1·74), assuming *D* = 2 days, and 2·23 (2·10–2·36), 2·37 (2·30–2·46), 2·33 (2·21–2·45), and 2·37 (2·26–2·48), assuming *D* = 4 days. These results are comparable to previous estimates made using whole population mortality.^5^,^16^ *R*_e_s estimated based on the two threshold definitions (i.e. stricter and broader) were not significantly different (paired *t*-test, *P* = 0·180 and 0·501 for the first two waves, respectively). Statistical tests indicate that *R*_e_ over the first 7 days were not significantly different between the four pandemic waves (anova test, *P* = 0·291). Similar to results for the entire population, if *R*_e_ is calculated over a 14-day period, the results are significantly lower than when calculated over the 7-day period (for 2nd wave, one-sided paired *t*-test, *P* = 2·2e-16). Over a 14-day period, *R*_e_ estimates for the second and third waves were 1·42 (1·40–1·45) and 1·35 (1·32–1·37), assuming *D* = 2 days, and 1·85 (1·81–1·89) and 1·69 (1·64–1·74), assuming *D* = 4 days. The estimates for the third wave were significantly lower than those for the second wave (one-sided *t*-test, *P* = 5·331e-06). We did not calculate the values for the first and final waves, due to the briefer duration of these waves (<14 days) for many 1-year age cohorts.

To look for potential trends of *R*_*e*_ versus age, we did a simple linear regression for each wave. The correlation was not statistically significant for the first three waves (*P* = 0·285, 0·538, and 0·532, respectively) and slightly significant for the fourth (*P* = 0·026, adjusted *r*^2^ = 0·096). These results indicate that there generally was no systematic age-specific difference of *R*_e_ among different age cohorts during the pandemic.

## Discussion

Using daily NYC mortality data, we have identified the timing and age-distributed mortality pattern of each 1918 pandemic wave among 1-year age-grouped cohorts. Although less accurate for assessing disease transmission dynamics due to variability in individual time to death,^13^ mortality records provide valuable information in the absence of detailed morbidity records. In contrast to other regions with a herald 1918 wave during spring-summer outside the regular flu season,^10^,^11^,^17^ the first wave in NYC appears to have begun during a time of year typically suitable for the transmission of influenza. The timing and transmissibility of influenza (including pandemic influenza) may be affected by environmental conditions such as ambient humidity.^18^–^20^ Typical low wintertime humidity levels should have created conditions favorable for the spread of influenza, and indeed, historical meteorological records indicate that humidity levels during February and March 1918 were low (data not shown). In fact, humidity levels were lower during the first pandemic wave than during the second and should have been conducive to the rapid transmission of influenza in a fully susceptible population. Yet, despite the presence of a supposedly fully naïve population and favorable humidity conditions, the first wave appears to have been muted.

The first pandemic wave closely followed a seasonal influenza outbreak in NYC. People recently infected with this seasonal influenza would likely have had increased titers of antibodies for this seasonal strain. One possible explanation for the limited extent of the first pandemic wave is that individuals with those high seasonal influenza antibody titers benefitted from partial protection against the pandemic strain, despite any structural dissimilarities among these two influenza viruses. Indeed, short-lived non-strain-specific immunity has been proposed to occur following influenza infection and modify the attack rates of both seasonal and pandemic influenza.^21^,^22^ This partial immunity could have afforded protection for the population equivalent to herd immunity. Thus, the first pandemic wave could have been restricted among subpopulations with higher risk of infection, in particular, school-aged children, who have more frequent contact among peers and usually experience earlier and higher attack rates during both influenza epidemics and pandemics.^13^,^14^,^23^,^24^ Indeed, our analysis indicates that school-aged children had the highest fractional mortality increase during the first wave. As cross-immunity waned in the months following winter,^25^ population susceptibility to the pandemic strain might have increased substantially so that by the onset of the second wave, an unchecked outbreak with severe morbidity and mortality had been enabled.

An analysis of the 1918 summer and fall waves in Copenhagen suggests that the CFR in the summer wave was many folds lower than fall (˜0·3% versus 2·3%).^10^ The idea that the virus obtained increased pathogenicity after having obtained broad transmissibility contradicts evolution theory.^26^ Instead, if recently boosted immunity effectors (e.g. antibodies) from previous infections were able to provide partial cross-protection or down-regulate immunity specific to the pandemic strain, they might have reduced the chance of the ‘cytokine storm’ that is believed to cause most influenza-related deaths in young adults.^27^ This hypothesis may provide an alternative explanation for the less severe mortality of the first wave in NYC and for the lower CFR in the first wave in Copenhagen.

The unusual age distribution of morbidity and mortality patterns during the pandemic is also noteworthy. Surveys conducted during 1918–1919 suggest that young adults (20–39 years of age) suffered high morbidity as well as CFR; persons 5–19 years of age had the highest morbidity but lowest CFR.^7^,^14^ In contrast, persons >˜50 years of age had the lowest morbidity, and a CFR was comparable to interpandemic seasons (Figures 2 and 3 in ref. 3). Generally, our analysis on NYC mortality records agrees with these prior findings. In addition, a recent study by Viboud *et al*.^28^ found a minimum excess mortality risk at 9–10 years of age and a maximum at 24–26 years of age in fall 1918 in Kentucky. Here, we show that the fractional excess mortality dipped and peaked around the same age groups for NYC. This consistency suggests that these age-specific mortality patterns were not random.

It has been suggested that exposure to a former circulating strain among the older population provided specific partial immunoprotection.^2^,^3^ Even so, this suggestion cannot fully explain the differing CFR among children versus young adults who both had no exposure to this previously circulating strain. As concluded in Viboud *et al*.,^28^ this atypical age mortality patterns likely result from a combination of unknown factors.

Based on ILI case reports over 2–4 weeks, Chowell *et al*.^29^ estimated *R*_e_ for the three waves during the 2009 pandemic in Mexico; they showed that *R*_e_ decreased from 1·8–2·1 in the spring to 1·6–1·9 in the summer and further to 1·2–1·3 in the fall (*D* = 3 and 4 days). The authors attributed this decline in *R*_e_ to higher levels of herd immunity and better intervention measures in the later waves. Likewise, we found that *R*_e_ over the first 2 weeks was greater at the second wave than at the third one. However, our analyses on mortality data over the first 7 days reveal that the *R*_e_s at the first week of the final two pandemic episodes were comparable to those in the first two waves.

Due to the size of NYC and potential isolation of communities within the city, some subpopulations may have remained largely unaffected by early waves of the pandemic. It is possible that later waves of the pandemic were fomented through introduction of the virus into these naïve communities. Therefore, at the very beginning of the later episodes, the pandemic could still grow at a pace comparable to the earlier waves. As susceptibles from these subpopulations depleted in the following weeks, particularly during the later waves, the wave then attenuated to a lower growth rate. Clearly, modern community structure differs tremendously from that of 1918; our society is now unprecedentedly interconnected. Nevertheless, heterogeneity among populations still exists and clusters of susceptibles may still be major targets during the later phase of an epidemic or pandemic. If so, more prevention measures (e.g. vaccination) should be allocated to communities with lower influenza prevalence. Further analysis of the heterogeneity of morbidity and mortality among the population may provide more insight into these ideas.

This study has several limitations. We used all-cause mortality data rather than more specific P&I data for this analysis. For the major pandemic waves (i.e. the second and third), these two mortality data sets were comparable (differing only by a factor of 1·02^8^); in contrast, as shown in Olson *et al*.,^8^ excess all-cause mortality was 1·42 and 1·37 times higher than excess P&I mortality during the first and last waves, respectively. These differences may reflect increased attribution of influenza-related mortality to other causes during the weaker first and last waves when the pandemic was novel and trailing off, respectively. We thus used all-cause mortality to more comprehensively capture excess mortality during each of the pandemic waves.

Secondly, pandemic-related mortality was estimated by subtracting the median baseline mortality, which during the winter included deaths from seasonal influenza. This metric thus may have underestimated the total mortality in the last two pandemic episodes. To address this problem, an excess seasonal influenza-related mortality could be added to the last two pandemic episodes.

Thirdly, due to a lack of detailed demographic data during the 1918 pandemic, we scaled the mortality increase by the baseline mortality for each age group, rather than the population of that group. The premise is that the baseline mortality would roughly scale with the population size of each age. This assumption is most valid for age groups with similar all-cause mortality rates, that is, likely those ˜20–40 years of age.^30^ Therefore, our approach is more accurate for comparing the age patterns of adults. Due to the higher baseline mortality associated with very young children (<5 years), their fractional mortality increases were lower than those of other groups, despite having a high absolute mortality increase (Table 1). Nevertheless, previous studies of grouped data clearly have shown that young adults 20–40 years of age suffered the most mortality during the 1918 pandemic,^3^,^8^ in corroboration with the current findings.

Additionally, we did not consider the age structure in the population when estimating *R*_e_. Rather, *R*_e_ was estimated assuming the pandemic spread independently within each age group. While this assumption of independence is unrealistic, potential imbalance in the transmissibility among age groups may manifest as higher *R*_e_ in certain age groups and should be detectable through our simple analysis. For instance, school children are usually assumed to have high *R*_e_ due to more frequent contact within their age group. However, our estimation suggests that there was no significant difference in *R*_e_ among age groups for the 1918 pandemic.

In conclusion, we have identified the calendar periods of the 1918 pandemic waves with improved precision, illustrated the demographic patterns of mortality within these episodes, and quantified the transmission characteristics for all four pandemic waves in NYC.

## Supporting Information

**Figure S1**. Age specific excess mortality attributable to each pandemic wave.

**Figure S2.** Mortality time series for each 1-year age cohort in the first pandemic episodes.

**Figure S3**. Mortality time series for each 1-year age cohort in the second pandemic episodes.

**Figure S4**. Mortality time series for each 1-year age cohort in the third pandemic episodes.

**Figure S5**. Mortality time series for each 1-year age cohort in the fourth pandemic episodes.

**Figure S6.** Calendar periods of the four pandemic episodes identified by using double the median daily baseline year mortality as the pandemic threshold.

**Figure S7.** Calendar periods of the four pandemic episodes identified by a threshold of 1·8 times of median daily baseline year mortality.

**Figure S8.** Calendar periods of the four pandemic episodes identified by a threshold of 1·5 times of median daily baseline year mortality.

**Figure S9.** Comparing statistics of daily mortality in the baseline years.

**Appendix S1.** Supplemental methods and discussion.

## References

[1] Johnson NP, Mueller J (2002). Updating the accounts: global mortality of the 1918–1920 “Spanish” influenza pandemic. Bull Hist Med.

[2] Morens DM, Fauci AS (2007). The 1918 influenza pandemic: insights for the 21st century. J Infect Dis.

[3] Taubenberger JK, Morens DM (2006). 1918 Influenza: the mother of all pandemics. Emerg Infect Dis.

[4] Reid AH, Taubenberger JK (2003). The origin of the 1918 pandemic influenza virus: a continuing enigma. J Gen Virol.

[5] Mills CE, Robins JM, Lipsitch M (2004). Transmissibility of 1918 pandemic influenza. Nature.

[6] Bureau of Labor Statistics http://www.bls.gov/opub/uscs/.

[7] Frost WH (2006). The epidemiology of influenza. 1919. Public Health Rep.

[8] Olson DR, Simonsen L, Edelson PJ, Morse SS (2005). Epidemiological evidence of an early wave of the 1918 influenza pandemic in New York City. Proc Natl Acad Sci USA.

[9] Sheng ZM, Chertow DS, Ambroggio X (2011). Autopsy series of 68 cases dying before and during the 1918 influenza pandemic peak. Proc Natl Acad Sci USA.

[10] Andreasen V, Viboud C, Simonsen L (2008). Epidemiologic characterization of the 1918 influenza pandemic summer wave in Copenhagen: implications for pandemic control strategies. J Infect Dis.

[11] Chowell G, Viboud C, Simonsen L, Miller MA, Acuna-Soto R (2010). Mortality patterns associated with the 1918 influenza pandemic in Mexico: evidence for a spring herald wave and lack of preexisting immunity in older populations. J Infect Dis.

[12] Germann TC, Kadau K, Longini IM, Macken CA (2006). Mitigation strategies for pandemic influenza in the United States. Proc Natl Acad Sci USA.

[13] Brundage JF, Shanks GD (2008). Deaths from bacterial pneumonia during 1918–19 influenza pandemic. Emerg Infect Dis.

[14] Britten R (1932). The incidence of epidemic influenza, 1918–1919. Public Health Rep.

[15] United States Department of Health and Human Services http://www.flu.gov/pandemic/history/1918/your_state/northeast/newyork/index.html.

[16] He DH, Dushoff J, Day T, Ma JL, Earn DJD (2011). Mechanistic modelling of the three waves of the 1918 influenza pandemic. Theor Ecol.

[17] Chowell G, Bettencourt LM, Johnson N, Alonso WJ, Viboud C (2008). The 1918–1919 influenza pandemic in England and Wales: spatial patterns in transmissibility and mortality impact. Proc Biol Sci.

[18] Shaman J, Goldstein E, Lipsitch M (2011). Absolute humidity and pandemic versus epidemic influenza. Am J Epidemiol.

[19] Shaman J, Kohn M (2009). Absolute humidity modulates influenza survival, transmission, and seasonality. Proc Natl Acad Sci USA.

[20] Yang W, Elankumaran S, Marr LC (2012). Relationship between humidity and influenza A viability in droplets and implications for influenza's seasonality. PLoS ONE.

[21] Ferguson NM, Galvani AP, Bush RM (2003). Ecological and immunological determinants of influenza evolution. Nature.

[22] Mathews JD, McBryde ES, McVernon J, Pallaghy PK, McCaw JM (2010). Prior immunity helps to explain wave-like behaviour of pandemic influenza in 1918–9. BMC Infect Dis.

[23] Lemaitre M, Carrat F (2010). Comparative age distribution of influenza morbidity and mortality during seasonal influenza epidemics and the 2009 H1N1 pandemic. BMC Infect Dis.

[24] Olson DR, Heffernan RT, Paladini M, Konty K, Weiss D, Mostashari F (2007). Monitoring the impact of influenza by age: emergency department fever and respiratory complaint surveillance in New York City. PLoS Med.

[25] Buchy P, Vong S, Chu S (2010). Kinetics of neutralizing antibodies in patients naturally infected by H5N1 virus. PLoS ONE.

[26] Flint SJ, Enquist LW, Racaniello VR, Skalka AM (2004). Evolution and emergence. Principles of Virology, Molecular Biology, Pathogenesis, and Control of Animal Viruses.

[27] Kobasa D, Takada A, Shinya K (2004). Enhanced virulence of influenza A viruses with the haemagglutinin of the 1918 pandemic virus. Nature.

[28] Viboud C, Eisenstein J, Reid AH, Janczewski TA, Morens DM, Taubenberger JK (2013). Age- and sex-specific mortality associated with the 1918–1919 influenza pandemic in Kentucky. J Infect Dis.

[29] Chowell G, Echevarría-Zuno S, Viboud C (2011). Characterizing the epidemiology of the 2009 influenza A/H1N1 pandemic in Mexico. PLoS Med.

[30] The New York City Department of Health and Mental Hygiene http://www.nyc.gov/html/doh/downloads/pdf/vs/1961sum.pdf.

