# Supplementary material for: The 1918 influenza pandemic in New York City: age-specific timing, mortality, and transmission dynamics
Source: Influenza Other Respir Viruses. 2013 Dec 2;8(2):177–88. doi: 10.1111/irv.12217 (PMC4082668; doi:10.1111/irv.12217)
Supplement: Supplementary file 1 — Figure S1. Age specific excess mortality attributable to each pandemic wave. [file irv0008-0177-SD1.docx]

Figure S1. Age specific excess mortality attributable to each pandemic wave. The number associated with each data point denotes the age at the time of each wave.
