# Supplementary material for: The 1918 influenza pandemic in New York City: age-specific timing, mortality, and transmission dynamics
Source: Influenza Other Respir Viruses. 2013 Dec 2;8(2):177–88. doi: 10.1111/irv.12217 (PMC4082668; doi:10.1111/irv.12217)
Supplement: Supplementary file 2 — Figure S2. Mortality time series for each 1-year age cohort in the first pandemic episodes. [file irv0008-0177-SD2.docx]

Figure S2 Mortality time series for each 1-year age cohort in the first pandemic episodes. The black dots denote daily mortality, and those within the identified pandemic period were connected with a red (by the stricter threshold definitions) and/or a blue (by the looser threshold definitions) line. The green lines show the threshold.
