# Supplementary material for: The 1918 influenza pandemic in New York City: age-specific timing, mortality, and transmission dynamics
Source: Influenza Other Respir Viruses. 2013 Dec 2;8(2):177–88. doi: 10.1111/irv.12217 (PMC4082668; doi:10.1111/irv.12217)
Supplement: Supplementary file 6 — Figure S6. Calendar periods of the four pandemic episodes identified by using double the median daily baseline year mortality as the pandemic threshold. [file irv0008-0177-SD6.docx]

Figure S6 Calendar periods of the four pandemic episodes identified by using double the median daily baseline year mortality as the pandemic threshold. Labels on the x-axis are dates (mm/dd). The numbers associated with the end of each segment are ages at the time of each pandemic episode.
