# Supplementary material for: The 1918 influenza pandemic in New York City: age-specific timing, mortality, and transmission dynamics
Source: Influenza Other Respir Viruses. 2013 Dec 2;8(2):177–88. doi: 10.1111/irv.12217 (PMC4082668; doi:10.1111/irv.12217)
Supplement: Supplementary file 8 — Figure S8. Calendar periods of the four pandemic episodes identified by a threshold of 1·5 times of median daily baseline year mortality. [file irv0008-0177-SD8.docx]

Figure S8 Calendar periods of the four pandemic episodes identified by a threshold of 1.5 times of median daily baseline year mortality. Labels on the x-axis are dates (mm/dd). The numbers associated with the end of each segment are ages at the time of each pandemic episode.
