# Supplementary material for: The 1918 influenza pandemic in New York City: age-specific timing, mortality, and transmission dynamics
Source: Influenza Other Respir Viruses. 2013 Dec 2;8(2):177–88. doi: 10.1111/irv.12217 (PMC4082668; doi:10.1111/irv.12217)
Supplement: Supplementary file 9 — Figure S9. Comparing statistics of daily mortality in the baseline years. [file irv0008-0177-SD9.docx]

Figure S9 Comparing statistics of daily mortality in the baseline years. The mean, median, highest, and 2^nd^ highest daily mortality were plotted for Age 4-9 as examples.
