# Supplementary material for: The 1918 influenza pandemic in New York City: age-specific timing, mortality, and transmission dynamics
Source: Influenza Other Respir Viruses. 2013 Dec 2;8(2):177–88. doi: 10.1111/irv.12217 (PMC4082668; doi:10.1111/irv.12217)
Supplement: Supplementary file 10 — Appendix S1. Supplemental methods and discussion. [file irv0008-0177-SD10.docx]

**Supplementary Material**

**The 1918 influenza pandemic in New York City: Age-specific timing, mortality, and transmission dynamics**

Wan Yang, Elisaveta Petkova, Jeffrey Shaman

**Mortality Data**

Death certificates prior to 1949 are stored at the New York City Department of Records and Information Services. All death indices, including day, month, year, borough, and age of each documented death between 1915 and 1923 in all New York City boroughs (Bronx, Brooklyn, Manhattan, Queens, and Staten Island), were scanned by the Genealogy Federation of Long Island. Permission to scan these records was obtained from Commissioner Brian Anderson.

All scanned records were entered into an electronic spreadsheet and subsequently proof read and edited to ensure accuracy. We merged and converted all records into a database where each of the fields is stored in a consistent format. Where possible, age information recorded in various formats (e.g. 50 ½ years, ¾ months, stillborn) was converted to integers rounded to the lower value. Records containing ambiguous information such as unrealistic numeric values for date or age, and spelling errors such as extra symbols or spaces, were eliminated. Records containing missing values were also eliminated.

We calculated the average annual number of deaths from the daily mortality data for each year from 1915 to 1923 and compared them to the annual number of reported deaths. Annual numbers of reported deaths during the study period were obtained from New York City’s Bureau of Vital Statistics. Annual calculated number of deaths was between 0.29% and 2.97% (median 1.78%) higher than the reported. The difference may be due to reporting criteria employed by the agency.

**Time series of mortality for each 1-year age cohort within each identified pandemic episode.**

To examine the pandemic periods identified by our definitions of onset and ending, we plotted time series of age-specific mortality for each pandemic episode (Figures S1-S4). The black dots denote daily mortality, and those within the age-specific identified pandemic period were connected with a red (by the stricter threshold definitions) and/or a blue (by the looser threshold definitions) line. The green lines show the threshold.

Based on the mortality time series for the entire NYC population, there were four pandemic waves occurring roughly within the following periods: 2/15 to 6/1/1918 (1^st^ wave), 8/1 to 12/2/1918 (2^nd^ wave), 12/3/1918 to 4/30/1919 (3^rd^ wave), and 12/1/1919 to 4/30/1920 (4^th^ wave). Within each of these periods, for each 1-year age cohort, we searched for the first 7 consecutive day timespan with mortality exceeding the estimated 90% quantile threshold level (i.e., onset) and the final day of the last 7 consecutive day timespan with mortality exceeding the same threshold (i.e., ending). All days between the onset and ending were included as a pandemic wave specific for each 1-year age cohort.

Ideally, a pandemic wave would persist from the onset through the ending with mortality continuously exceeding the defined threshold, and this is generally true for the second wave when the pandemic led to tremendous mortality increase. However, over the course of the first, third, and fourth waves, there were windows of time (e.g., several consecutive days) when the excess mortality dropped below the threshold. Therefore, for these waves, some weeks in the midst of onset through ending had more than 2 days with mortality below the threshold.

**Selecting the threshold for identifying the onset and ending of a pandemic episode.**

We tested a number of threshold for identifying the onset and ending of each pandemic episode. The Serfling method is commonly applied to estimate a non-influenza baseline morbidity or mortality.[^1-3^](#_ENREF_1) Further, Olson et al.[^4^](#_ENREF_4) defined an influenza season epidemic (including a pandemic wave) as two or more consecutive months with observed pneumonia & influenza (P&I) mortality exceeding the upper 95% confidence limit of the Serfling regression baseline. However, our attempt using this approach suggested that it is less suitable for processing daily data. The baseline fitted by the Serfling method is a fixed sinusoidal curve that does not account for random noise in the data well.

Instead, we tested thresholds defined in terms of quantiles of the baseline daily mortality data. A number of definitions, described below, were tested. All threshold definitions were examined by searching for the onset and ending of each pandemic wave in the following periods: 2/15 to 6/1/1918 (1^st^ wave), 8/1 to 12/2/1918 (2^nd^ wave), 12/3/1918 to 4/30/1919 (3^rd^ wave), and 12/1/1919 to 4/30/1920 (4^th^ wave).

(1) Median baseline daily mortality

We first tested thresholds defined as median baseline daily mortality multiplied by a factor of either 2, 1.8, or 1.5. Results showed that multiplication with a factor of 2 is too large and misses much of the pandemic signal at the beginning and ending of each pandemic wave, especially for the first, third, and fourth waves (Fig. S5). Lowering the factor to 1.5 or 1.8 allowed better capture of the beginning and ending of each pandemic wave in most age groups (Fig. S6 and S7). However, it appeared to include more noise. For instance, with these thresholds, the second pandemic wave is defined to start during early August 1918 for several groups (i.e., ages 12, 25, 29, and 43). This early onset arises because median daily mortalities for these age groups are near 0 during summer, and thus the defined thresholds are themselves only nominally above 0. As a consequence, small increases of mortality during summer, some of which may represent noise, may be misidentified as the onset of a wave. Due to these shortcomings, thresholds were not defined relative to baseline median.

(2) Maxima of daily baseline mortality

To account for the extreme values in daily mortality data, we then tested a threshold defined as the highest daily baseline mortality. However, this threshold was found to be too extreme and when used underestimates pandemic wave duration (Fig. S8).

(3) The second highest daily baseline mortality

With data for 6 baseline years, we only had limited empirical discrimination of quantiles (i.e., 8.33%, 25.00%, 41.67%, 58.33%, 75.00%, 91.67%) for each calendar day. Given the variations in mortality on any day over different years and the apparent over-extremeness of the maxima, we thus selected the second highest among the 6 baseline years as the daily baseline cut-off. We then set the pandemic threshold as 120% of this cut-off to better approximate the 90% quantile (Figs. 2 in the main text and Figs. S1-4).

In addition, this threshold was validated using the entire NYC population; excess daily mortality on the onset of each wave (i.e., 51, 70, 106, and 59 excess mortality, for a population of 5.6 million in NYC[^5^](#_ENREF_5)) was comparable to the threshold adopted by Mills et al.[^6^](#_ENREF_6) (i.e., 1 excess P&I mortality per 100,000 population). The value for the third wave was higher, probably due to the overlap of the second and third waves.

**Discussion on the shift of mortality age patterns over different pandemic waves.**

In the main text, we report that the fractional mortality increase (i.e., ratio of excess mortality to baseline mortality) was highest among teenagers during the first wave but shifted to 25-29 year olds in subsequent waves. This shift stems from a large increase in young adult excess mortality and is not an artifact of wave-to-wave changes in age-specific baseline mortality (the denominator). Indeed, the excess mortality difference between young adults and teenagers rises from a factor of ~2 during the first wave to ~4-6 in the second through fourth waves (Fig. S1).

One may argue that this shift is limited to the mortality data and may not extend to attack rates due to age-related differences in CFR. Lower CFRs have been reported for teenagers based on historical surveys[^8^](#_ENREF_8)^,^[^9^](#_ENREF_9) during Fall 1918 and Winter 1919. Our analysis shows that age-specific mortality patterns were highly correlated among the last three waves (Figure 4); these correlations suggest that the 1918 pandemic strain disproportionally caused higher CFR in certain age groups (i.e., age 20-29). It thus seems reasonable to infer that the 1918 pandemic strain might have produced a similar CFR in each age group during the first NYC pandemic wave. If the age-specific CFRs in the first wave were indeed similar to those in the subsequent waves, the higher fractional mortality increase shown for the teenagers during the first wave would manifest due to much higher attack rates than in other age groups.

Historical morbidity records are not available to confirm that 1918 pandemic attack rates were higher among school children in the spring wave but then shifted to more general population in subsequent waves. However, our analysis based on mortality records and existing age-specific CFR records during multiple waves of the 1918 pandemic does lend support to this inference. In addition, such alternation in age specific morbidity (and mortality) patterns has been observed for other outbreaks. Indeed, higher attack rates have been observed during the early period of more recent, and better documented, influenza pandemics.[^10^](#_ENREF_10)^,^[^11^](#_ENREF_11)

**References:**

1. Chowell G, Viboud C, Simonsen L, Miller MA, Acuna-Soto R. Mortality patterns associated with the 1918 influenza pandemic in Mexico: evidence for a spring herald wave and lack of preexisting immunity in older populations. Journal of Infectious Diseases 2010;202:567-75.

2. Andreasen V, Viboud C, Simonsen L. Epidemiologic characterization of the 1918 influenza pandemic summer wave in Copenhagen: implications for pandemic control strategies. Journal of Infectious Diseases 2008;197:270-8.

3. Serfling RE, Sherman IL, Houseworth WJ. Excess pneumonia-influenza mortality by age and sex in three major influenza A2 epidemics, United States, 1957-58, 1960 and 1963. Am J Epidemiol 1967;86:433-41.

4. Olson DR, Simonsen L, Edelson PJ, Morse SS. Epidemiological evidence of an early wave of the 1918 influenza pandemic in New York City. Proceedings of the National Academy of Sciences of the United States of America 2005;102:11059-63.

5. Bureau of Labor Statistics. 100 Years of U.S. Consumer Spending: Data for the Nation, New York City, and Boston. <http://www.bls.gov/opub/uscs/> Accessed May 8, 2013, 2013.

6. Mills CE, Robins JM, Lipsitch M. Transmissibility of 1918 pandemic influenza. Nature 2004;432:904-6.

7. The New York City Department of Health and Mental Hygiene. Summary of vital statistics 1961: the City of New York. <http://www.nyc.gov/html/doh/downloads/pdf/vs/1961sum.pdf> Accessed 8/12/2013, 2013.

8. Britten R. The incidence of epidemic influenza, 1918-1919. Public Health Reports 1932;47:303-339.

9. Frost WH. The epidemiology of influenza. 1919. Public Health Reports 2006;121 Suppl 1:149-59; discussion 148.

10. Glezen WP. Emerging infections: pandemic influenza. Epidemiologic Reviews 1996;18:64-76.

11. Lemaitre M, Carrat F. Comparative age distribution of influenza morbidity and mortality during seasonal influenza epidemics and the 2009 H1N1 pandemic. BMC Infect Dis 2010;10:162.

**Figure Legends**

Fig. S1 Age specific excess mortality attributable to each pandemic wave. The number associated with each data point denotes the age at the time of each wave.

Figs. S2-5 Mortality time series for each 1-year age cohort in the four pandemic episodes. The black dots denote daily mortality, and those within the identified pandemic period were connected with a red (by the stricter threshold definitions) and/or a blue (by the looser threshold definitions) line. The green lines show the threshold.

Fig. S6 Calendar periods of the four pandemic episodes identified by using double the median daily baseline year mortality as the pandemic threshold. Labels on the x-axis are dates (mm/dd). The numbers associated with the end of each segment are ages at the time of each pandemic episode.

Fig. S7 Calendar periods of the four pandemic episodes identified by a threshold of 1.8 times of median daily baseline year mortality. Labels on the x-axis are dates (mm/dd). The numbers associated with the end of each segment are ages at the time of each pandemic episode.

Fig. S8 Calendar periods of the four pandemic episodes identified by a threshold of 1.5 times of median daily baseline year mortality. Labels on the x-axis are dates (mm/dd). The numbers associated with the end of each segment are ages at the time of each pandemic episode.

Fig. S9 Comparing statistics of daily mortality in the baseline years. The mean, median, highest, and 2^nd^ highest daily mortality were plotted for Age 4-9 as examples.
